# St. Bartholomew's Hospital

**Published:** 1904-11-05

**Authors:** 


### ST. BARTHOLOMEW'S HOSPITAL.

THE following letter from Sir Trevor Lawrence appeared in the *Times* of October 27th in reply to the articles which were published in the *Daily Mail* of October 22nd and also in *Truth*:—

"I should be grateful if you would find space to insert a few particulars as to the progress of the appeal for the rebuilding of St. Bartholomew's Hospital made to the public at a meeting at the Mansion House last January.

"The response which the governors have so far received amounts to £84,000. This in no way discourages them, for they regard it as a considerable sum to have raised during a year of exceptional trade depression, although it is but a small proportion of the sum required for the completion of our rebuilding scheme.

"The governors propose, in the first instance, to carry out the absolutely necessary work of building the block which is to provide a new casualty and out-patient department, the latter to comprise adequate accommodation for ophthalmic, orthopædic, skin, throat, ear, dental, electrical, diseases of women, and other special departments.

"The foundation-stone of this block was laid by his Majesty the King in July last; and it will be remembered that, in giving his cordial approval to the proposals of the governors, his Majesty expressed his belief that the public would not allow this beneficent undertaking to be hindered by the want of the necessary funds.

"This block will cost upwards of £100,000.

"In view of the misapprehension as to the policy and plans of the governors, which appears to prevail in certain quarters, it may be well to state that such repairs as have been carried out in the existing buildings this year have been of a strictly limited character, the principal outlay being less than £1,000 for the electric lighting of the west wing of the hospital, the necessity of which was admitted on all hands.

"The governors are fully alive to the importance of ultimately achieving a complete reconstruction of the hospital buildings. In particular they are anxious to provide, as soon as possible, a pathological department, new mortuary, and post-mortem rooms, and a nurses' home. The accommodation for the nurses, as at present existing, is of a makeshift character, and they are housed, for the most part, in old and inadequate buildings.

"It must not be supposed that the governors have in any way departed from the intention they have repeatedly expressed of reconstructing the hospital in its entirety in accordance with the views of their medical staff. But this can only be done prudently as funds come in.

"The critics of St. Bartholomew's have overlooked the fact that the committee, appointed by the late Lord Mayor to consider the question, resolved, by 15 to one, that it was essential, in the interests of the public, that the hospital should be rebuilt on the site which it had occupied for close on 800 years.

"The governors feel that, in carrying out the policy adopted by the almost unanimous verdict of what was in great measure an independent body, they have the right to appeal for help to the charitable public and especially to those who are indebted to their connection with the City for prosperity and wealth.

"The history of St. Bartholomew's, and the work it has done during many hundreds of years, are too well known to need recapitulation here; but I would beg leave to impress upon your readers the urgency of helping this ancient institution to adapt itself to the circumstances and needs of the present day, without impairing its completeness and efficiency by largely diminishing its income."

"It is but right to remind those who, in the interests of other institutions, blame the governors for not postponing their appeal that a single year's postponement means a sacrifice of £9,000 interest and sinking fund on the cost of the land bought from Christ's Hospital."

St. Bartholmew's Hospital, October 25th.

---

The *Westminster Gazette* of the 27th ult. publishes the following interview:—

THE FUTURE OF "BART'S."

"A *Westminster* representative had a conversation yesterday with Mr. Andrew Motion, who is a governor of the hospital and a member of the House Committee. That gentleman had only just heard of Sir Henry Burdett's suggestion that he should become the head of affairs at Bart's.

"'I would do anything to see St. Bartholomew's placed upon a sound footing,' said Mr. Motion. 'I live 100 miles from London, but I would gladly give up a year of my life if I could do any good. I agree that a stringent administrative reform is absolutely necessary. At present it is little less than a farce my attending meetings of the House Committee. It is difficult to obtain any information as to what is being done. Last week I attended a meeting at which it was proposed to grant the retiring secretary, Mr. Cross, a pension of £900 a year. I, without in any way questioning the possibility that Mr. Cross might, for his services, deserve this large pension, asked for particulars to show how the amount was arrived at. I got no particulars, nor was my request backed up by any other member. I hear now that the pension has been granted. It does, indeed, seem a large amount to grant, when the hospital is appealing in vain to the public for funds.

"'Our income is about £90,000 a year, but before that is free to be applied to the practical purposes of the hospital it has been diminished in one way and another by £30,000. It seems ridiculous, and I think nothing will meet the present muddle but a properly organised commission of inquiry, or a royal commission, if necessary.'"

